# Prevalence of *Trichinella* spp. Infections in Hunted Wild Boars in Northern Iran

**Published:** 2017-12

**Authors:** Ali ROSTAMI, Hooshang KHAZAN, Bahram KAZEMI, Eshrat Beigom KIA, Mojgan BANDEPOUR, Niloofar TAGHIPOUR, Gholamreza MOWLAVI

**Affiliations:** 1. Dept. of Parasitology and Mycology, School of Medicine, Shahid Beheshti University of Medical Sciences, Tehran, Iran; 2. Dept. of Biotechnology, School of Medicine, Shahid Beheshti University of Medical Sciences, Tehran, Iran; 3. Dept. of Medical Parasitology & Mycology, School of Public Health, Tehran University of Medical Sciences, Tehran, Iran

**Keywords:** Wild boar, Meat, Human trichinellosis, Iran

## Abstract

**Background:**

Trichinellosis is an important and neglected foodborne zoonotic infectious disease in worldwide. The most human outbreaks in recent years have been related to consumption of wild boar meat. This cross-sectional study determined the prevalence of *Trichinella* spp. infections in hunted wild boars in northern Iran.

**Methods:**

Thirty-five hunted wild boars were subjected in this study in 2015. All samples were examined by conventional artificial digestion method to detect of muscle larvae. Genomic DNA was extracted by phenol-chloroform method from isolated larvae. To identify the *Trichinella* species, a PCR-based method was applied using the internal transcribed spacer 2 (ITS2) and mitochondrial small-subunit ribosomal RNA (rRNA) gene sequences.

**Results:**

The overall prevalence of *Trichinella* spp. infection was 5.7% (2/35, 95%CI= 0–13.4). The mean larval burdens in two positive samples were 0.05 and 6 larvae per gr tissue muscle, respectively. The PCR reaction, using specific primers, yielded two 367 bp and 195 bp bands on agarose gel for ITS 2 and rrnS, respectively.

**Conclusion:**

There is a hidden burden of *Trichinella* spp. infection in wild boar population in Iran. Moreover, *T. britovi* is the prevalent species circulating in wild boars of Iran. Therefore, education of the hunters and other consumers should be performed about the risk of consumption of raw or undercooked meat and meat products from wild boars.

## Introduction

Human trichinellosis, a foodborne zoonotic infectious disease, caused by consumption of raw or inadequately processed meat or meat products containing encysted muscle larvae of the nematode worms of the genus *Trichinella* (1). Human infection with *Trichinella* spp. has been reported in 55 countries of the world with an average of 5751 cases and five deaths per year (2–4). The global number of disability-adjusted life years (DALYs) due to trichinellosis be 76 per billion persons per year (95% credible interval: 38–129) (2). The course of human infection can be divided into two phases including an intestinal phase and a muscular phase. The main clinical symptoms are diarrhea and abdominal pain in first stage (intestinal phase) and fever, myalgia, myocarditis, skin allergenic reactions and encephalitis in second stage (muscular phase) (1, 5).

The pork’s meat is considered as a major source of human infection, but meat from wild boars, horses, bears, badgers, dogs, and walruses, also plays an important role (3, 6). The wild boars, important source of trichinellosis, are indigenous in many countries, and recent outbreaks due to consumption of their meat have been reported in worldwide (1, 7–10). These outbreaks indicated potential role of wild boars as a reservoir for human infection and demonstrated that contaminated meat products from a single infected wild boar carcass can infect hundreds of people at risk of disease if it is not well cooked before consumption (1).

In Iran, wild boar population is growing rapidly in recent years, mainly regarding the religious believes based on not consuming pork and wild boar meat. Therefore, hunting of wild boar is forbidden in Iran except for religious minorities (e.g., Jews, Christians, Zoroastrians, etc.). However, there is illegal hunting and consumption of wild boar meat even among Muslim populations (observational data). Moreover, there are two published reports of human trichinellosis following consumption of wild boar meat in Iran (11, 12).

In Iran, little is known about the prevalence of *Trichinella* spp. infection in wild boar and people level risks of the meat consumption. Therefore, the main objective of the present study was to determine the prevalence of *Trichinella* spp. infections in hunted wild boars in northern Iran. The result of this study is useful for estimating the risk of exposure to these parasites from wild boars for humans in Iran.

## Materials and Methods

### Study area

This preliminary cross-sectional study was performed in three cities (Neka, Amol, and Chalus) in Mazandaran Province, Northern Iran, from Jan 2015 to Feb 2016. This area (36.5° 25′N 53° 21′E) has a hot-summer Mediterranean climate, a mean annual temperature of 16 °C and about 900 mm of precipitation falls annually (13). This area is an endemic area for many parasitic infections (13) and is a popular area for hunting wild boars for Iranian hunters and foreign tourists.

### Parasitological examination

Totally, 35 hunted wild boars (15 Amol, 12 Neka, and 8 Chalus) were subjected for determination of *Trichinella* spp. infection. Muscle samples from shoulders, posterior legs, tongue, and diaphragm were collected from each carcass and transported to the helminthology laboratory at the Department of Parasitology and Mycology, Shahid Beheshti University of Medical Sciences, Tehran, Iran. From each wild boar, at least 60 gr of muscles were digested by artificial digestion magnetic stirrer method in 1% HCl, 1% pepsin and sedimented according to a previously described protocol (14). Following artificial digestion, a stereomicroscopic (Zeiss, Germany) examination based on morphological characteristics was performed to identify the *Trichinella* larvae. Isolated *Trichinella* larvae were stored in 70% ethyl alcohol at −20 °C for molecular identification.

### Ethical statement

All procedures in this study were approved by the Ethics Committee of the Shahid Beheshti University of Medical Science, before the beginning of the study.

### DNA extraction from larvae

Total genomic DNA was extracted using the conventional phenol-chloroform method as described previously with some modification (15). Briefly, a pool of approximately 30 muscle larvae of *Trichinella* was placed in a 1.5 ml microcentrifuge tube and mixed with 500 μl of lysis buffer (50 mM Tris-HCl pH 8.0, 200 mM NaCl, 20 mM EDTA pH 8.0 and 1% SDS). Following sonication using a T10 UltraTurrax tissue disperser (IKA-WERK, Staufen, Germany), 10-μl proteinase K (20 mg/ml; Qiagen) was added and the mixture was incubated at 55 °C for overnight. Proteinase K was inactivated at 100 °C for 30 min. Purification of DNA was done by phenol and phenol-chloroform extraction, followed by ethanol precipitation. DNA concentrations were determined by spectrophotometrically by GeneQuant 100 (GE Healthcare Life Science). DNA was resuspended with 30 μl distilled water and frozen at −20 °C for use in PCR amplifications.

### Polymerase chain reaction amplification

A 195 bp fragment of the mitochondrial small subunit of the ribosomal RNA (rRNA) was amplified by PCR with two primers TRICH rrnS F (5′-CATGGTTAGGTGAGATATTGCCTGC-3′) and TRICH rrnS R (5′-GGTCCTCCTTCCAGAAGATCTACTTTG-3′) previously reported (16). The PCR reactions were performed in a 15-μl reaction containing 7.5 μl of master mix (Ampliqon, Denmark), 10 pmol of primer F and R, 2 μl of extracted DNA and 4.5 μl of distilled water. Reactions were amplified for 35 cycles in a thermocycler (Techne, England) under the following conditions: denaturation at 94 °C for 30 sec, annealing at 62 °C for 30 sec, and elongation at 72 °C for 30 sec. Initial denaturation and final extension were at 94 °C for 5 min and 72 °C for 5 min, respectively.

The primers to amplify a 367 bp region of the internal transcribed spacer 2 (ITS2) gene of *Trichinella* (BritI 5′-AAAACCGGTGAGCGTAATAAAG-3′ and BritII 5′-CGAGCGCCTAACACCACAATA-3′) were derived from those described by Mayer-Scholl et al. (17). For the amplification of ITS2 gene, PCR reaction, initial denaturation, and final extension were same to above and amplification was carried out for 35 cycles, each consisting of 60 sec at 94 °C, 30 sec at 58 °C, and 30 sec at 72 °C. After staining with ethidium bromide, PCR products were electrophoresed on 1.5% and 2% agarose gel for ITS2 and rrnS, respectively. The amplicons were visualized under UV illumination.

### DNA sequence analysis

PCR amplicons were sequenced in using Applied Biosystems 3730/3730xl DNA Analyzers (Bioneer, Korea). All sequences were assembled and edited manually using the Chromas program ver. 1.0.0.1. Basic Local Alignment Search Tool (BLAST; http://blast.ncbi.nlm.nih.gov) was used to analyze sequences obtained from this study against data in GenBank. To determine the nucleotide sequence diversity of both genes, we used of online Multalin (*multalin.toulouse.inra.fr/multalin/*) and BioEdit (ver. 7.0.0) software.

### Statistical analysis

The frequency and percentage were used to describe the prevalence of *Trichinella* spp. in wild boars. Overall, 95% confidence intervals (CI) were calculated using binomial distribution.

## Results

**Fig. 1:**
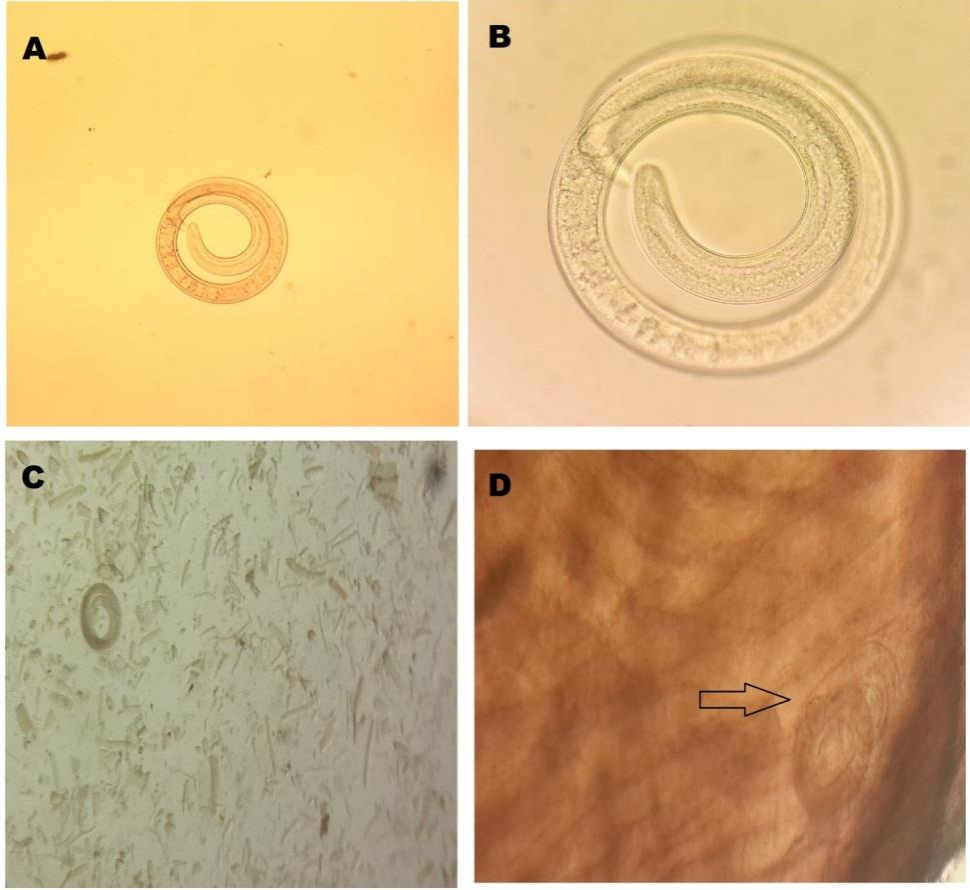
A–C larvae of *Trichinella* spp. in juice of digested wild boar meat; D) larvae of *Trichinella* spp. in diaphragm

Across the 35 tested wild boars and using both digestion and molecular methods, the overall prevalence of *Trichinella* spp. larvae infection was 5.7% (2/35, 95% CI= 0–13.4). Both infected wild boar were from Amol county (2/15, 13.3%). The mean larval burdens in two positive samples were 0.05 and 6 larvae per gram tissue muscle, respectively (Fig. 1 A–D).

**Fig. 2:**
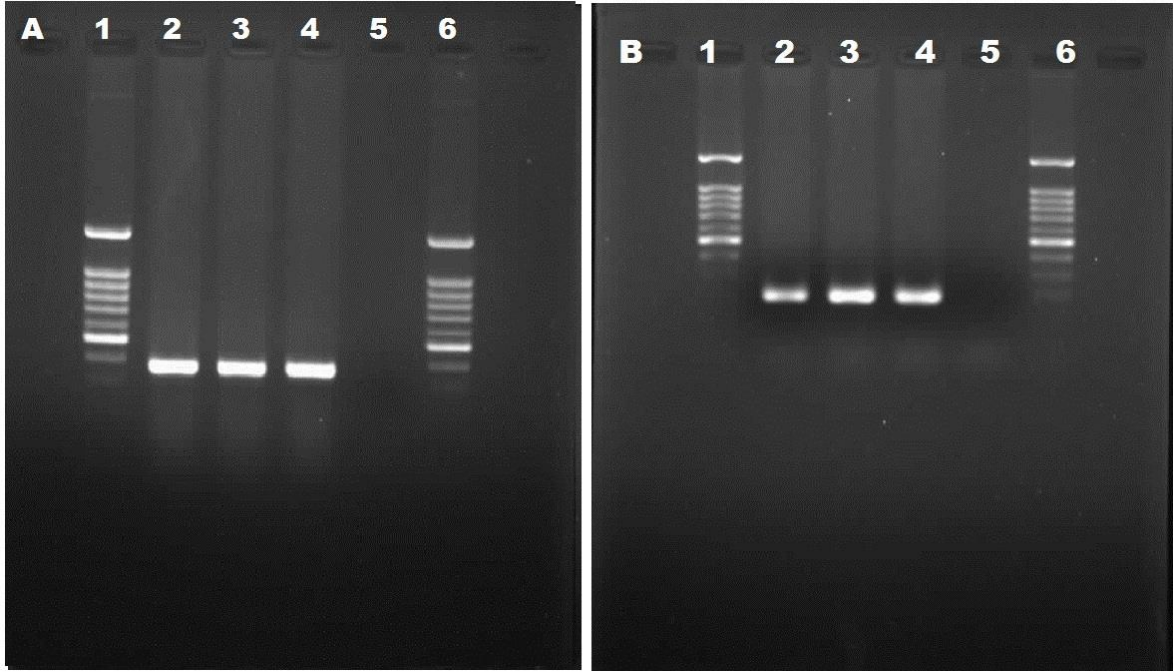
Diagnostic amplification of the *Trichinella* spp. A and B) Lan 2, 3, DNA samples amplified with internal transcribedspacer 2 (ITS2) gene amplimer pairs [367 bp] (A) and rrnS amplimer pairs [195 bp] (B); Lan 4, positive control sample; Lan 5, negative control; Lan 1 and 6, DNA Marker (100 bp)

*Trichinella* spp. larvae in muscles were visible inside thick collagen capsules by direct microscopic (Fig. 1D). The PCR reaction, using specific primers, yielded two 367 bp and 195 bp bands on agarose gel for ITS2 and rrnS, respectively (Fig. 2 A, B).

**Fig. 3:**
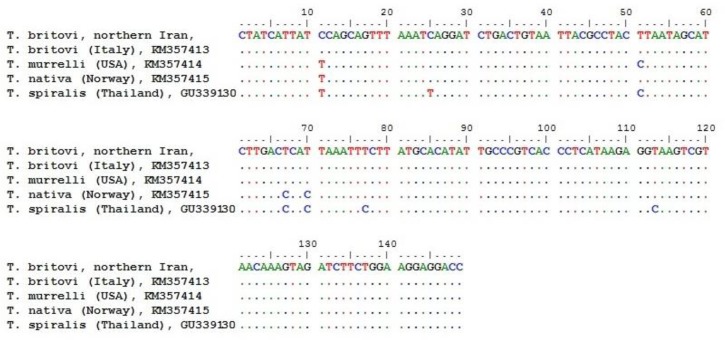
Sequence alignment of isolates from this study against reference sequences in GenBank according to rrns gene

Sequence analyses revealed that both positive isolates were *T. britovi*. BLAST analysis of rrns gene showed 100% homology with those of *T. britovi* available in GenBank (accession nos. KM357413.1 and DQ159092.1), whereas homology with the *T. murrelli* (KM357414.1), *T. native* (KM357415.1) and *T. spiralis* (GU339130.1) reference sequences was 99%, 98%, and 95%, respectively. Nucleotide sequence diversities for rrns gene are shown in Fig. 3.

**Fig. 4:**
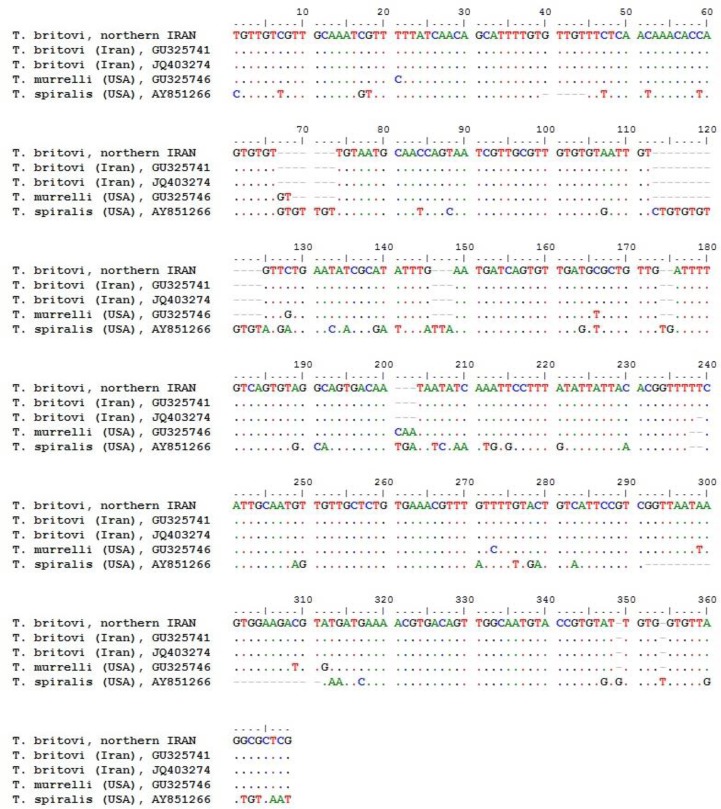
Sequence alignment of isolates from this study against reference sequences in GenBank according to ITS2 gene

BLAST analysis of ITS2 gene demonstrated 100% and 99% homology with *T. britovi* GU325741.1 and JQ403274.1 reference sequences, respectively. Homology with the *T. murrelli* (GU325746.1) and *T. spiralis* (AY851266.1) reference sequences was 96% and 79%, respectively. Nucleotide sequence diversities of isolates from this study against reference sequences available in GenBank regarding ITS2 gene are presented in Fig. 4.

## Discussion

There is a dramatically decline in human trichinellosis during the second half of the 20th century following improvement in pig rearing practices, preventive medicine, and disease control programs in the USA and Europe (18). Parallel to the decrease in sources of human infection among domestic animals in recent years (pig and horse), there is an increasing attention to the distribution of *Trichinella* spp. infection in wildlife and most human outbreaks in recent years have been related to consumption of wild boar meat (1, 8, 9, 18).

The potential role meat and meat-derived products from wild boar as the second source of human trichinellosis is well documented in literature (3, 18). In fact, hunter and their friends consumed wild boar meat locally without proper cooking. Moreover, epidemiological data from some outbreaks showed that sausages made with meat from domestic pigs might be mixed with sausages made with contaminated meat from infected wild boar (18).

The result obtained from this study revealed a 5.7% prevalence of *Trichinella* spp. infection in wild boar populations in northern Iran, confirming the presence of a great threat to acquisition of infection among hunters and another consumer of wild boar meat in this area. Although we found *Trichinella* spp. infection in only two carcasses, but as mentioned in introduction, consumption of contaminated meat products even from a single infected wild boar carcass can infect hundreds of people, such as recent outbreak in Germany (1). The prevalence of *Trichinella* spp. infection in our study (5.7%) is higher than previously conducted studies in Mazandaran (0.04%, 2/4,950) and Golestan (0.02%, 5/21,143) provinces in northern Iran, and is lower than study performed in Khuzestan Province (0.25, 1/4) in southwest of Iran (19, 20). However, these studies were carried out about 40 years ago, and recent studies in Iran are very rare. In one recent study in northeast of Iran, *Trichinella* spp. infection was not found among 25 hunted wild boars (21). In Islamic communities, the consumption of wild boar meat is forbidden. However, several outbreaks are reported in some of them (22). There are two previous reports on occurrence of human trichinellosis in Iran following consumption of wild boar meat. The first human case was reported in 1966 due to the consumption of meat from a wild boar in Golestan Province (12), east to Mazandaran Province. Since then, only one outbreak, consisting of 6 cases, was reported in 2007 due to ingestion of raw wild boar meat hunted in Gilan Province, northern Iran (11), west to Mazandaran Province. However, in both report, definitive diagnoses were based on clinical finding and serological tests (11, 12). Although, our field observation indicates that a relatively significant Iranian population (families and friends of both religious minorities and Muslims hunters) are consumer of wild boar meat and a remarkable number of wild boars are hunted annually. Therefore, rate of infection be relatively remarkable, but since symptomology of trichinellosis in healthy individuals is mild and also awareness about it is very little, many of infected cases are overlooked.

Our results showed that *T. britovi* is circulated in wild boar populations in north of Iran. This finding is in line with recent molecular studies to identifying of *Trichinella* isolated from wild animals in Iran (21, 23, 24). *T. britovi* had been identified in a leopard (*Panthera pardus* saxicolor) from the Ardabil Province, north-western Iran, two stray dogs from the Khorasan Razavi Province, north-eastern Iran and in two jackals (*Canis aureus*) from the Khuzestan Province, south-west Iran (21, 23, 24). Moreover, isolates from this study demonstrated 100% homology with *T. britovi* responsible for the reported latest outbreak in Iran (25). Although in 1983 and in the absence of molecular techniques, *Trichinella* sp. Was isolated from four Iranian golden jackals and identified them as *T. spiralis* (one isolate from the Mazandaran Province) and as *T. nelsoni* (three isolates from the Khuzestan Province) by cross-breeding experiments (26). Today, the *T. nelsoni* isolates from the Palaearctic region are named *T. britovi* (24, 27, 28).

## Conclusion

There is a hidden burden of *Trichinella* spp. infection in wild boars population in Iran that could be a neglected public health problem. Therefore, education of the hunters and other consumers should be performed about the risk of consumption of raw or undercooked meat and meat products from wild boars. Moreover, additional preventive measures to reduce the risk the transmission of *Trichinella* spp. to domestic animals are necessary for Iran.

## Ethical considerations

Ethical issues (Including plagiarism, informed consent, misconduct, data fabrication and/or falsification, double publication and/or submission, redundancy, etc.) have been completely observed by the authors.
